# Epidemiology of Myocarditis and Pericarditis Following mRNA Vaccination by Vaccine Product, Schedule, and Interdose Interval Among Adolescents and Adults in Ontario, Canada

**DOI:** 10.1001/jamanetworkopen.2022.18505

**Published:** 2022-06-24

**Authors:** Sarah A. Buchan, Chi Yon Seo, Caitlin Johnson, Sarah Alley, Jeffrey C. Kwong, Sharifa Nasreen, Andrew Calzavara, Diane Lu, Tara M. Harris, Kelly Yu, Sarah E. Wilson

**Affiliations:** 1 Public Health Ontario, Ontario, Canada; 2 Dalla Lana School of Public Health, University of Toronto, Toronto, Ontario, Canada; 3 ICES, Toronto, Ontario, Canada; 4 Department of Family and Community Medicine, University of Toronto, Toronto, Ontario, Canada; 5 University Health Network, Toronto, Ontario, Canada

## Abstract

**Importance:**

Increased rates of myocarditis or pericarditis following receipt of COVID-19 mRNA vaccines have been observed. However, few available data are associated with differences in rates of myocarditis or pericarditis specific to vaccine products, which may have important implications for vaccination programs.

**Objective:**

To estimate rates of reported myocarditis or pericarditis following receipt of a COVID-19 mRNA vaccine by product, age, sex, dose number, and interdose interval.

**Design, Setting, and Participants:**

This population-based cohort study was conducted in Ontario, Canada (population: 14.7 million) from December 2020 to September 2021 and used data from Ontario’s COVID-19 vaccine registry and passive vaccine-safety surveillance system. All individuals in Ontario, Canada, who received at least 1 dose of COVID-19 mRNA vaccine between December 14, 2020, and September 4, 2021, and had a reported episode of myocarditis or pericarditis following receipt of the COVID-19 vaccine during this period were included. We obtained information on all vaccine doses administered in the province to calculate reported rates of myocarditis or pericarditis.

**Exposures:**

Receipt of a COVID-19 mRNA vaccine (mRNA-1273 [Moderna Spikevax] or BNT162b2 [Pfizer-BioNTech Comirnaty]).

**Main Outcomes and Measures:**

All reports of myocarditis or pericarditis meeting levels 1 to 3 of the Brighton Collaboration case definitions were included. Rates and 95% CIs of reported cases of myocarditis or pericarditis per 1 000 000 mRNA vaccine doses administered were calculated by age, sex, dose number, vaccine product, and interdose interval.

**Results:**

Among 19 740 741 doses of mRNA vaccines administered, there were 297 reports of myocarditis or pericarditis meeting the inclusion criteria; 228 (76.8%) occurred in male individuals, and the median age of individuals with a reported event was 24 years (range, 12-81 years). Of the reported cases, 207 (69.7%) occurred following the second dose of the COVID-19 mRNA vaccine. When restricted to individuals who received their second dose during the period of enhanced passive surveillance (on or after June 1, 2021), the highest rate of myocarditis or pericarditis was observed in male individuals aged 18 to 24 years following mRNA-1273 as the second dose (299.5 cases per 1 000 000 doses; 95% CI, 171.2-486.4 cases per 1 000 000 doses); the rate following BNT162b2 as the second dose was 59.2 cases per 1 000 000 doses (95% CI, 19.2-138.1 cases per 1 000 000 doses). Overall rates for both vaccine products were significantly higher when the interdose interval was 30 or fewer days (BNT162b2: 52.1 cases per 1 000 000 doses [95% CI, 31.8-80.5 cases per 1 000 000 doses]; mRNA-1273: 83.9 cases per 1 000 000 doses [95% CI, 47.0-138.4 cases per 1 000 000 doses]) compared with 56 or more days (BNT162b2: 9.6 cases per 1 000 000 doses [95% CI, 6.5-13.6 cases per 1 000 000 doses]; mRNA-1273: 16.2 cases per 1 000 000 doses [95% CI, 10.2-24.6 cases per 1 000 000 doses]).

**Conclusions and Relevance:**

The findings of this population-based cohort study of Ontario adolescents and adults with myocarditis or pericarditis following mRNA COVID-19 vaccination suggest that vaccine products and interdose intervals, in addition to age and sex, may be associated with the risk of myocarditis or pericarditis after receipt of these vaccines. Vaccination program strategies, such as age-based product considerations and longer interdose intervals, may reduce the risk of myocarditis or pericarditis following receipt of mRNA vaccines.

## Key Points

**Question:**

Do rates of reported myocarditis or pericarditis following COVID-19 mRNA vaccination vary by vaccine product and interdose interval?

**Findings:**

This population-based cohort study of 297 individuals in Ontario, Canada, with myocarditis or pericarditis following COVID-19 vaccination found higher rates of myocarditis or pericarditis associated with receipt of mRNA-1273 compared with BNT162b2 as a second dose, particularly among male individuals aged 18 to 24 years. Higher rates were also observed with shorter interdose intervals.

**Meaning:**

The results suggest that there may be product-specific differences in rates of myocarditis or pericarditis after receiving mRNA vaccines and that programmatic strategies may be associated with reduced risk of myocarditis or pericarditis after receiving mRNA vaccines.

## Teaser

This cohort study estimates rates of myocarditis and pericarditis after receipt of an mRNA COVID-19 vaccine among individuals in Ontario, Canada, stratified by recipients’ age and sex and by the vaccine product, schedule, and interdose interval.

## Introduction

Postmarket vaccine-safety surveillance systems in multiple countries have identified a likely association of myocarditis or pericarditis with receipt of BNT162b2 (Pfizer-BioNTech Comirnaty) or mRNA-1273 (Moderna Spikevax) COVID-19 mRNA vaccines.^1,2,3,4,5^ In Ontario, Canada (with a population of approximately 14.7 million), enhanced surveillance for myocarditis or pericarditis following receipt of mRNA vaccines began in early June 2021. This surveillance comprised health care professional communication from the provincial government and public health agency, hospital-led algorithms for clinical investigations and management, and instructions on reporting events to the passive vaccine-safety surveillance system. This enhanced surveillance directive coincided with a number of changes to Ontario’s COVID-19 vaccination program, including expansion of vaccine eligibility to young adults and adolescents (Health Canada authorized BNT162b2 for individuals aged 12-15 years on May 5, 2021), a large acceleration in second-dose administration due to increased vaccine supply, permissive language from Canada’s National Advisory Committee on Immunization with regard to heterologous mRNA vaccine schedules (first and second doses with different vaccines),^6^ and, during summer 2021, a gradual return to scheduling second doses in accordance with product monograph intervals following a period of extended intervals between the first and second doses (ie, interdose intervals) that had been used to maximize the number of individuals protected with a first dose of vaccine.^6^ These programmatic changes provided an opportunity to examine the risk of myocarditis or pericarditis in association with a number of factors. Preliminary analyses reported in August 2021 were limited in scope.^7^

Our objective was to examine rates of reported myocarditis or pericarditis following mRNA vaccination by age, sex, vaccine product, dose number, interdose interval, and homologous (first and second doses with the same vaccine) or heterologous vaccine schedule using passive vaccine-safety surveillance data.

## Methods

In this cohort study conducted from December 2020 to September 2021, we used the Public Health Case and Contact Management Solution, the electronic reporting system in Ontario, Canada for COVID-19 adverse events following immunization, to identify myocarditis and pericarditis after COVID-19 vaccination reported between December 14, 2020 (start of the vaccination program), and September 4, 2021. In Ontario, the reporting of adverse events following immunization by health care professionals is mandated by legislation; voluntary reporting by vaccine recipients or caregivers also occurs.^8^ Reports are submitted to local public health units, which conduct additional investigations and obtain supporting information (eg, laboratory findings and diagnostic imaging). The Public Health Ontario Ethics Review Board determined that this study did not require research ethics committee approval or informed consent because the study activities were conducted in fulfillment of Public Health Ontario’s legislated mandate “to provide scientific and technical advice and support to the health care system and the Government of Ontario in order to protect and promote the health of Ontarians” (Ontario Agency for Health Protection and Promotion Act, S.O. 2007, C. 10) and were therefore considered public health practice, not research. We followed the Strengthening the Reporting of Observational Studies in Epidemiology (STROBE) reporting guidelines for cohort studies.^9^

Events were identified through a keyword search (ie, “myocarditis” or “pericarditis”) or if cardiovascular injury, myocarditis, or pericarditis was selected from a list of predefined adverse events. A case-level review of all reports was completed by specialized nurses and physicians on Public Health Ontario’s vaccine safety team to assign a level of diagnostic certainty using Brighton Collaboration (BC) case definitions for myocarditis and pericarditis.^10^ The BC case definitions for myocarditis and pericarditis have been adopted by the Public Health Agency of Canada for passive vaccine-safety surveillance activities.^11,12^ We restricted our analyses to events meeting BC levels 1 to 3 of diagnostic certainty. In sensitivity analyses that examined only myocarditis, reports of adverse events following immunization with physician diagnoses of myocarditis, myopericarditis, or perimyocarditis were included only if the BC case definition (levels 1-2) for myocarditis was met. We included all reports following vaccination, regardless of time since vaccination, in crude rates. We obtained information on receipt of vaccines from the provincial COVID-19 vaccine registry, COVaxON.

### Statistical Analysis

We calculated rates and 95% CIs of reported cases of myocarditis or pericarditis per 1 000 000 mRNA vaccine doses administered by age, sex, dose number, and vaccine product for strata with at least 1 event. The 95% CIs were calculated using the Poisson exact method. Our primary analysis was restricted to individuals who initiated their vaccine series on or after June 1, 2021, to account for potential increases in reporting of adverse events following immunization after heightened awareness resulting from media reports and the provincial enhanced surveillance directive for myocarditis or pericarditis that began in early June 2021. This timing coincided with other changes to the vaccination program, including implementation of heterologous mRNA schedules (eFigure 1 in the Supplement). We repeated these analyses for sensitivity analyses that included myocarditis only and both myocarditis and pericarditis for the period from December 14, 2020, to September 4, 2021.

We also calculated these rates stratified by homologous or heterologous vaccine schedule and by interdose interval, restricted to individuals who received their second dose (regardless of the first dose date) on or after June 1, 2021, to maximize our sample of second-dose recipients during the period of enhanced surveillance. We selected the interval groupings by examining the distribution of intervals among individuals receiving a second dose and to align with the product monographs and programmatic decisions (ie, extended interdose intervals). We assessed statistical significance using nonoverlapping 95% CIs. We repeated this analysis for male individuals aged 18 to 24 years given the large number of reported events in this age group.

To compare the rates following the second dose by vaccine product, we also used Poisson regression to calculate age- and sex-stratified rate ratios and 95% CIs, adjusting for first-dose product and interdose interval. Data were analyzed using SAS Enterprise Guide, version 8.2 (SAS Institute). All tests were 2-sided and used a significance level of *P* < .05.

## Results

Between December 14, 2020, and September 4, 2021, there were 19 740 741 doses of mRNA vaccines administered in Ontario and 417 cases of myocarditis or pericarditis reported to the provincial system on adverse events following immunization. Of these cases, 297 (71.2%) met the inclusion criteria based on the BC case definitions (level 1-3); among these cases, 207 (69.7%) occurred following the second dose of COVID-19 mRNA vaccine, and 228 (76.8%) occurred in male individuals. The median age among individuals meeting BC level 1 to 3 criteria was 24 years (range, 12-81 years) (Table 1). Events were classified as myopericarditis (107 [36.0%]), followed by myocarditis (105 [35.4%]) and pericarditis (85 [28.6%]). Nearly all events (290 [97.6%]) involved an emergency department visit, with 210 events (70.7%) also requiring hospital admission. The number of individuals hospitalized was 87 (82.9%) for myocarditis, 33 (38.8%) for pericarditis, and 90 (84.1%) for myopericarditis (eTable 1 in the Supplement). The time to symptom onset was available for 295 cases (99.3%), and the median time to onset among these individuals was 3 days after vaccination (IQR, 2-8 days; range, 0-73 days). Most events (218 [73.9%]) with a known onset date occurred within 7 days of vaccine administration. For events following a second dose, 179 (86.9%) occurred within 7 days of vaccination, and 200 (97.1%) occurred within 30 days (eFigure 2 in the Supplement).

**Table 1.** Characteristics of Myocarditis and Pericarditis Reports After COVID-19 mRNA Vaccines

Characteristic	Patients, No. (%)^a^				
	After first dose (n = 90)		After second dose (n = 207)		Total (N = 297)
	Administered before June 1	Administered on or after June 1	Administered before June 1	Administered on or After June 1	
Total reports, No.	50	40	5	202	297
Age, y					
Median (range)	32 (12-81)	23 (13-76)	50 (34-61)	23 (12-81)	24 (12-81)
12-17	5 (10.0)	14 (35.0)	0 (0.0)	36 (17.8)	55 (18.5)
18-24	12 (24.0)	7 (17.5)	0 (0.0)	77 (38.1)	96 (32.3)
25-39	11 (22.0)	10 (25.0)	2 (40.0)	49 (24.3)	72 (24.2)
≥40	22 (44.0)	9 (22.5)	3 (60.0)	40 (19.8)	74 (24.9)
Sex					
Female	18 (36.0)	10 (25.0)	3 (60.0)	38 (18.8)	69 (23.2)
Male	32 (64.0)	30 (75.0)	2 (40.0)	164 (81.2)	228 (76.8)
Time to onset, median (IQR), d^b^	15 (7-29)	4 (2-14)	2 (2-73)	2 (1-3)	3 (2-8)
Vaccine product					
BNT162b2	39 (78.0)	29 (72.5)	4 (80.0)	87 (43.1)	159 (53.5)
mRNA-1273	11 (22.0)	11 (27.5)	1 (20.0)	115 (56.9)	138 (46.5)
Clinical diagnosis					
Myocarditis	18 (36.0)	13 (32.5)	2 (40.0)	72 (35.6)	105 (35.4)
Pericarditis	23 (46.0)	15 (37.5)	2 (40.0)	45 (22.3)	85 (28.6)
Myopericarditis^c^	9 (18.0)	12 (30.0)	1 (20.0)	85 (42.1)	107 (36.0)
Healthcare use or outcome					
Emergency department visit	49 (98.0)	37 (92.5)	5 (100.0)	199 (98.5)	290 (97.6)
In-patient hospitalization	32 (64.0)	24 (60.0)	4 (80.0)	150 (74.3)	210 (70.7)
Intensive care unit admission	1 (2.0)	3 (7.5)	0	10 (5.0)	14 (4.7)
Death	0	0	0	0	0

Abbreviations: BNT162b2, Pfizer-BioNTech Comirnaty; mRNA-1273, Moderna Spikevax.

^a^ Data are presented as the number (percentage) of patients unless otherwise indicated.

^b^ Two reports with unknown time to onset were excluded from this calculation.

^c^ Includes myocarditis or pericarditis (2 patients), myopericarditis (81 patients), and perimyocarditis (24 patients).

In our primary analysis focusing on those who initiated their vaccination series on or after June 1, 2021, the rate of myocarditis or pericarditis tended to be higher after the second dose of the mRNA vaccine than after the first dose, particularly for individuals who received mRNA-1273 as the second dose of the series (Table 2). The highest rate of myocarditis or pericarditis was observed among male individuals aged 18 to 24 years following mRNA-1273 as the second dose (299.5 cases per 1 000 000 doses [95% CI, 171.2-486.4 cases per 1 000 000 doses]) compared with BNT162b2 as the second dose (59.2 cases per 1 000 000 doses [95% CI, 19.2-138.1 cases per 1 000 000 doses]) in this age group. The second highest rate was observed among male individuals aged 12 to 17 years following their second dose of BNT162b2 (97.3 cases per 1 000 000 doses [95% CI, 60.3-148.8 cases per 1 000 000 doses]). In the sensitivity analysis restricted to myocarditis only, the observed patterns remained unchanged (eTable 2 in the Supplement). The results were also similar for myocarditis or pericarditis for the period from December 14, 2020, to September 4, 2021 (eTable 3 in the Supplement; rates following the second dose by age in years and by vaccine product are shown in eFigure 3 in the Supplement).

**Table 2.** Crude Rate of Reported Myocarditis or Pericarditis per Million Vaccine Doses Administered by Vaccine Product, Dose Number, Age, and Sex With Series Initiation on or After June 1, 2021

Vaccine	Reported No. of cases per 1 000 000 doses, No. (95% CI)^a^					
	All individuals		Female individuals		Male individuals	
	Dose 1	Dose 2	Dose 1	Dose 2	Dose 1	Dose 2
**BNT162b2**						
Age group, y						
12-17	27.3 (14.9-45.8)	54.4 (34.5-81.7)	20.1 (6.5-47.0)	9.7 (1.2-35.1)	34.2 (15.6-64.9)	97.3 (60.3-148.8)
18-24	17.9 (5.8-41.7)	44.3 (17.8-91.3)	7.9 (0.2-44.1)	27.4 (3.3-99.0)	26.2 (7.1-67.0)	59.2 (19.2-138.1)
25-39	13.0 (5.2-26.8)	16.0 (5.2-37.4)	3.9 (0.1-21.6)	19.7 (4.1-57.6)	21.5 (7.9-46.7)	12.6 (1.5-45.4)
≥40	5.9 (1.2-17.3)	NR	4.0 (0.1-22.3)	NR	7.8 (0.9-28.3)	NR
Total	15.6 (10.4-22.4)	29.0 (20.2-40.3)	8.9 (3.9-17.6)	11.9 (4.8-24.5)	21.8 (13.5-33.3)	45.3 (30.1-65.5)
**mRNA-1273**						
Age group, y						
12-17^b^	NA	NA	NA	NA	NA	NA
18-24	21.6 (2.6-77.9)	195.5 (117.7-305.3)	NR	69.1 (14.2-201.9)	37.2 (4.5-134.6)	299.5 (171.2-486.4)
25-39	16.2 (3.3-47.3)	58.7 (30.3-102.6)	NR	21.5 (2.6-77.7)	28.8 (5.9-84.3)	90.1 (43.2-165.7)
≥40	30.0 (11.0-65.2)	NR	22.0 (2.7-79.4)	NR	36.7 (10.0-93.9)	NR
Total	23.0 (11.5-41.1)	62.5 (42.4-88.6)	9.5 (1.1-34.2)	22.0 (7.1-51.4)	33.7 (15.4-64.0)	96.8 (63.2-141.9)

Abbreviations: BNT162b2, Pfizer-BioNTech Comirnaty; mRNA-1273, Moderna Spikevax; NA, not applicable; NR, not reported.

^a^ Estimates were not provided for strata with 0 reported events.

^b^ Estimates for mRNA-1273 were not provided for individuals aged 12 to 17 years because this product was not used for this age group in Ontario.

To explore differences in the rate of myocarditis or pericarditis following the second dose of mRNA-1273 vs BNT162b2, we also examined rates by mixed schedule and interdose interval (Figure; additional data are shown in eTable 4 in the Supplement). Among all ages and sexes combined, rates of myocarditis or pericarditis were significantly higher for individuals with shorter interdose intervals for either vaccine received as the second dose (for intervals ≤30 days: BNT162b2, 52.1 cases per 1 000 000 doses [95% CI, 31.8-80.5 cases per 1 000 000 doses]; mRNA-1273, 83.9 cases per 1 000 000 doses [95% CI, 47.0-138.4 cases per 1 000 000 doses]; for intervals ≥56 days: BNT162b2, 9.6 cases per 1 000 000 doses [95% CI, 6.5-13.6 cases per 1 000 000 doses]; mRNA-1273, 16.2 cases per 1 000 000 doses [95% CI, 10.2-24.6 cases per 1 000 000 doses]) (Figure, A). This trend was also observed among male individuals aged 18 to 24 years across vaccine product combinations (eTable 5 in the Supplement). Overall, 95% CIs overlapped when comparing homologous with heterologous schedules within second-dose products.

**Figure.**
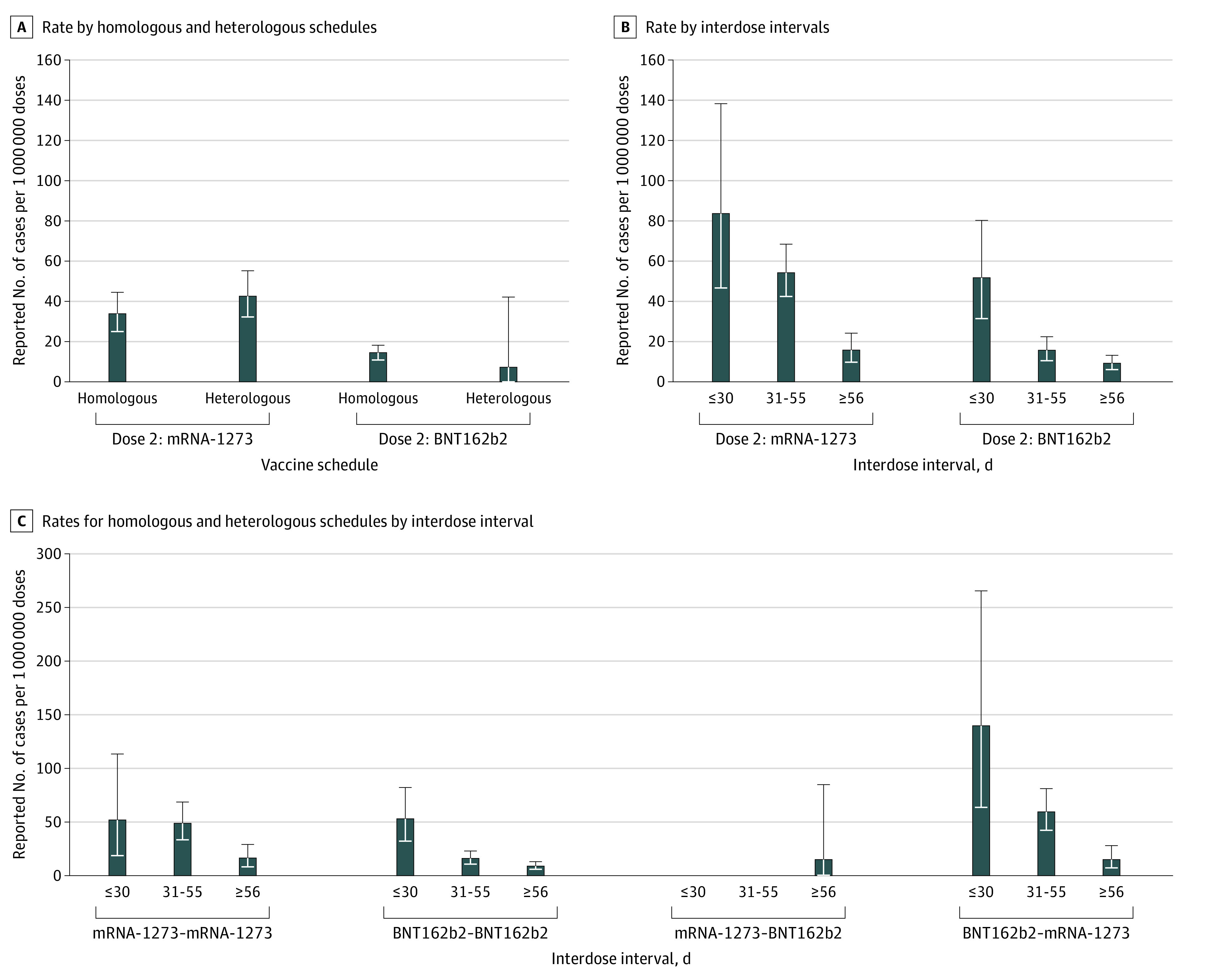
Overall Rate of Reported Myocarditis or Pericarditis Among People Who Completed the 2-Dose Vaccine Series With the Second Dose on or After June 1, 2021 Error bars indicate 95% CIs. BNT162b2, Pfizer-BioNTech Comirnaty; mRNA-1273, Moderna Spikevax.

Among individuals who received their second dose on or after June 1, 2021, adjusted rate ratios (aRRs) for myocarditis or pericarditis comparing mRNA-1273 with BNT162b2 were significantly higher among male individuals aged 18 to 24 years (aRR, 6.6; 95% CI, 3.3-13.2) and 25 to 39 years (aRR, 5.1; 95% CI, 2.3-11.5) and among female individuals aged 18 to 24 years (aRR, 9.6; 95% CI, 1.9-48.8) (Table 3). However, the 95% CIs in this analysis were wide owing to small numbers (ie, ≤10 events after a second mRNA dose among female individuals aged 18 to 24 years).

**Table 3.** Adjusted Rate Ratios for Myocarditis or Pericarditis, Comparing mRNA-1273 With BNT162b2 as the Second COVID-19 Vaccine Dose by Age and Sex Among Individuals Receiving Their Second Dose on or After June 1, 2021

Sex and age group, y	aRR (95% CI) for mRNA-1273 vs BNT162b2^a^	*P* value
Female		
18-24	9.6 (1.9-48.8)	.006
25-39	1.6 (0.4-6.3)	.51
≥40	0.5 (0.04-4.3)	.52
Male		
18-24	6.6 (3.3-13.2)	<.001
25-39	5.1 (2.3-11.5)	<.001
≥40	0.8 (0.3-2.7)	.76

Abbreviations: aRR, adjusted rate ratio; BNT162b2, Pfizer-BioNTech Comirnaty; mRNA-1273, Moderna Spikevax.

^a^ Adjusted for first dose product and interdose interval.

## Discussion

Using passive vaccine-safety surveillance data, we identified 297 cases of myocarditis or pericarditis that met the BC case definition following receipt of an mRNA vaccine since the start of the COVID-19 vaccination program in Ontario, Canada. Consistent with other surveillance systems and studies,^13,14^ we found that rates of myocarditis or pericarditis were highest among young male individuals following a second dose and that events were tightly clustered within the first week after vaccination. Rates were higher following a second dose of either mRNA-1273 or BNT162b2 compared with a first dose, and the rates following a second dose of mRNA-1273 were higher than those following a second dose of BNT162b2, in particular for young male individuals. In addition to product-specific insights for age or sex groups at highest risk of myocarditis or pericarditis after COVID-19 vaccination, our analyses suggest that interdose intervals and vaccine schedule combinations may also be associated with the risk of myocarditis or pericarditis. These observations suggest that there may be programmatic strategies in terms of vaccine product, interval, and schedule that may reduce the risk of myocarditis or pericarditis following receipt of mRNA vaccines.

The crude rates of reported cases of myocarditis and pericarditis after receipt of an mRNA COVID-19 vaccine in Ontario are consistent with estimates from other passive vaccine-safety surveillance systems and other data sources,^2,3,4,5,15^ although there is variability in age-specific rates across systems and countries. In Israel, where only BNT162b2 was used with a 21-day interdose interval, the rate of myocarditis (using the BC definition) following dose 2 among male individuals aged 16 to 19 years was 150 cases per 1 000 000 doses between December 2020 and May 2021, although this period included both active and passive surveillance periods.^2^ In the present study, the rate of myocarditis or pericarditis in Ontario among male individuals aged 12 to 17 years who received 2 doses of BNT162b2 at an interval of 30 days or less was similar at 159.7 cases per 1 000 000 doses. In the UK, the rate of reported cases of myocarditis after both the first and second doses across all ages was estimated at 10 cases per 1 000 000 doses of BNT162b2 and 36 cases per 1 000 000 doses of mRNA-1273 based on events submitted as of November 17, 2021,^4^ and for individuals aged 19 to 29 years, rates of myocarditis following dose 2 were 22 and 69 cases per 1 000 000 doses for BNT162b2 and mRNA-1273, respectively. This trend of an increased case rate after receiving mRNA-1273 is consistent with our findings, although the overall case rate in the UK study was lower than in our study. The UK used an extended interdose interval,^16^ and the overall results may be more comparable with those of our subgroup analyses examining rates among individuals with 8 or more weeks between doses. Rates across data sources in the US vary. With the use of data from 4 US Food and Drug Administration Biologics Effectiveness and Safety claims databases among male individuals aged 18 to 25 years, the rate of myocarditis or pericarditis within 7 days following a second dose of mRNA-1273 ranged from 72.4 cases per 1 000 000 doses (95% CI, 23.2-228.1 cases per 1 000 000 doses) to 283.7 cases per 1 000 000 doses (95% CI, 145.2-573.5 cases per 1 000 000 doses) across these 4 databases.^17^ In Ontario, we estimated a similar rate of myocarditis or pericarditis at 299.5 cases per 1 000 000 doses following a second dose of mRNA-1273 among male individuals aged 18 to 24 years. With the use of data from the Vaccine Adverse Event Reporting System, a passive reporting system, the rate of reported cases of myocarditis per 1 000 000 vaccine doses among male individuals with symptom onset within 7 days of a second dose of mRNA-1273 was much lower than estimated in the Biologics Effectiveness and Safety databases, at 38.5 cases per 1 000 000 doses.^18^ The case rate per 1 000 000 doses following a second dose of BNT162b2 in the Vaccine Adverse Event Reporting System data was 36.8 for male individuals aged 18 to 24 years, 69.1 for male individuals aged 16 to 17 years, and 39.9 for male individuals aged 12 to 15 years.^18^ Data from the US also include those from the active surveillance system Vaccine Safety Datalink, which has rates higher than in the Vaccine Adverse Event Reporting System.^19^ In a head-to-head analysis of BNT162b2 and mRNA-1273 among individuals aged 18 to 39 years, the Vaccine Safety Datalink reported that the adjusted rate of myocarditis or pericarditis within 7 days of dose 2 was 2.72 times greater (95% CI, 1.25-6.05 times greater) for those who received mRNA-1273 compared with BNT162b2, with an excess of 13.3 cases per 1 000 000 second doses of mRNA-1273 vs BNT162b2.^20^ There are several possible explanations for the differences in rates across systems, including outcomes studied (ie, myocarditis only vs myocarditis or pericarditis), time from vaccination to symptom onset for cases included in the analyses, different case definitions used to classify outcomes, completeness in reporting, and health system context (ie, access to publicly funded health services). In addition, our analyses suggest that country-specific differences in the interdose interval and heterologous vaccine schedules may also be associated with variability in rates across jurisdictions.

Following extensive review and discussion of the product-specific differences identified from passive vaccine-safety surveillance, Ontario modified its COVID-19 vaccine program on September 29, 2021, to preferentially offer BNT162b2 to individuals 12 to 24 years of age,^21^ which was later expanded up to 29 years of age following national guidance issued by Canada’s National Advisory Committee on Immunization.^22^ Although authorized by Health Canada in late August 2021 for adolescents 12 to 17 years of age, mRNA-1273 has yet to be incorporated into Ontario’s adolescent vaccination program. By mid-November 2021, several countries, including Norway, Sweden, Finland, France, and Germany, had issued similar guidance limiting the use of mRNA-1273 for adolescents and young adults.^23,24,25,26^

In addition, several immunization advisory bodies, including those in Canada, the US, Australia, and the UK, have issued guidance outlining the considerations for or providing clear recommendations to use longer intervals between primary-series vaccine doses with the dual aims of improving the durability of the immune response and possibly reducing the rare risk of myocarditis or pericarditis.^22,27,28,29^ The specific mechanism for this adverse event remains to be confirmed. Current etiologic hypotheses include a hyperimmune or hyperinflammatory response, delayed hypersensitivity, hypersensitivity to non-mRNA vaccine components, or an autoimmune response prompted by molecular mimicry, especially in individuals at increased risk due to hormonal or other (as yet unidentified) factors.^30,31^

Although data on the possible relative risks for myocarditis or pericarditis among vaccine products are emerging, these findings need to be considered within the context of absolute risk because myocarditis and pericarditis are still rare or very rare events, based on standard pharmacovigilance definitions.^32^ Of importance, the risk of myocarditis or pericarditis following receipt of mRNA vaccines also needs to be considered in association with risks of myocarditis following SARS-CoV-2 infection (ie, higher rates of myocarditis following infection than vaccination)^33,34,35^ and the high effectiveness of mRNA vaccine products.

This study’s analyses included data on all adverse events following immunization entered into a single passive vaccine-safety surveillance system in a large jurisdiction with high vaccine coverage (2-dose coverage among 77.6% of the vaccine-eligible population [ie, ≥12 years of age] as of September 4, 2021^36^). All reports of adverse events following immunization were individually reviewed by a team of specialized nurses and physicians to limit analyses to those events meeting BC case definitions for myocarditis or pericarditis (levels 1-3). We used data on the entire vaccination program through the provincial COVID-19 vaccine registry, which allowed us to examine rates of reported events in the context of detailed denominator data for various vaccine product schedules and intervals. This is helpful in assessing the mRNA vaccine program at the population level by interpreting the risk of myocarditis or pericarditis on the absolute scale (ie, cases per 1 000 000 vaccine doses administered) overall and by age, sex, dose number, and vaccine product. These results will also be helpful in the ongoing contextualization of the risk of myocarditis or pericarditis following receipt of mRNA vaccines compared with the risk of SARS-CoV-2 infection and associated outcomes.

### Limitations

This study has several limitations, including those inherent to passive vaccine-safety surveillance systems, such as stimulated reporting during the period of enhanced reporting. However, these limitations were minimized by restricting myocarditis or pericarditis events to only those meeting BC levels 1 to 3 and using thorough sensitivity analyses; when we analyzed rates in different time periods and restricted the analysis to myocarditis only, our conclusions were unchanged. In addition, several of our rates for product and schedule combinations were based on small numbers, leading to very wide 95% CIs; as such, rates for individual strata should be interpreted with caution.

## Conclusions

Although myocarditis or pericarditis following receipt of mRNA vaccines is rare, the findings of this study suggest that modifying mRNA COVID-19 vaccination programs to incorporate age-based product considerations and longer interdose intervals may reduce the risk of these events. Confirmation of these findings and further exploration of the association of heterologous mRNA vaccine schedules and interdose intervals with the risk of myocarditis or pericarditis are needed.

## Notes

Supplement. **eFigure 1.** Number of COVID-19 mRNA Vaccine Doses Administered in Ontario by Dose Number and Vaccine Product and Overview of Vaccine Program Changes **eFigure 2.** Myocarditis and Pericarditis Reports Following COVID-19 mRNA Vaccines by Dose Number and Time to Symptom Onset **eTable 1.** Clinical Diagnosis and Severity of Myocarditis and Pericarditis **eTable 2.** Crude Rate of Reported Myocarditis Meeting BC Level 1 and 2 Only per Million Doses Administered and 95% CI by Vaccine Product, Dose Number, Age, and Sex: Series Initiated on or After June 1, 2021 **eTable 3.** Crude Rate of Reported Myocarditis and Pericarditis per Million Doses Administered and 95% CI by Vaccine Product, Dose Number, Age, and Sex: Series Initiated on or After December 14, 2020 **eFigure 3.** Rate of Reported Myocarditis and Pericarditis per Million Doses Administered and 95% CI Following Dose 2 of mRNA Vaccine for All Sexes in Ontario as of September 4, 2021 **eTable 4.** Rate of Reported Myocarditis and Pericarditis per Million Doses Administered and 95% CI by Dose 2 Product and Interdose Interval, for Individuals Receiving Dose 2 on or After June 1, 2021 **eTable 5.** Rates of Reported Myocarditis and Pericarditis per Million Doses Administered and 95% CI Among Males Aged 18-24 Years by Vaccine Product and Interdose Interval With Dose 2 on or After June 1, 2021
