# Supplementary material for: Epidemiology of Myocarditis and Pericarditis Following mRNA Vaccination by Vaccine Product, Schedule, and Interdose Interval Among Adolescents and Adults in Ontario, Canada
Source: JAMA Netw Open. 2022 Jun 24;5(6):e2218505. doi: 10.1001/jamanetworkopen.2022.18505 (PMC9233237; doi:10.1001/jamanetworkopen.2022.18505)
Supplement: Supplement. — eFigure 1. Number of COVID-19 mRNA Vaccine Doses Administered in Ontario by Dose Number and Vaccine Product and Overview of Vaccine Program Changes eFigure 2. Myocarditis and Pericarditis Reports Following COVID-19 mRNA Vaccines by Dose Number and Time to Symptom Onset eTable 1. Clinical Diagnosis and Severity of Myocarditis and Pericarditis eTable 2. Crude Rate of Reported Myocarditis Meeting BC Level 1 and 2 Only per Million Doses Administered and 95% CI by Vaccine Product, Dose Number, Age, and Sex: Series Initiated on or After June 1, 2021 eTable 3. Crude Rate of Reported Myocarditis and Pericarditis per Million Doses Administered and 95% CI by Vaccine Product, Dose Number, Age, and Sex: Series Initiated on or After December 14, 2020 eFigure 3. Rate of Reported Myocarditis and Pericarditis per Million Doses Administered and 95% CI Following Dose 2 of mRNA Vaccine for All Sexes in Ontario as of September 4, 2021 eTable 4. Rate of Reported Myocarditis and Pericarditis per Million Doses Administered and 95% CI by Dose 2 Product and Interdose Interval, for Individuals Receiving Dose 2 on or After June 1, 2021 eTable 5. Rates of Reported Myocarditis and Pericarditis per Million Doses Administered and 95% CI Among Males Aged 18-24 Years by Vaccine Product and Interdose Interval With Dose 2 on or After June 1, 2021 [file jamanetwopen-e2218505-s001.pdf]

Supplementary Online Content

Buchan SA, Seo CY, Johnson C, et al. Epidemiology of myocarditis and pericarditis following mRNA vaccination by vaccine product, schedule, and interdose interval among adolescents and adults in Ontario, Canada. *JAMA Netw Open*. 2022;5(6):e2218505. doi:10.1001/jamanetworkopen.2022.18505

eFigure 1. Number of COVID-19 mRNA Vaccine Doses Administered in Ontario by Dose Number and Vaccine Product and Overview of Vaccine Program Changes

eFigure 2. Myocarditis and Pericarditis Reports Following COVID-19 mRNA Vaccines by Dose Number and Time to Symptom Onset

eTable 1. Clinical Diagnosis and Severity of Myocarditis and Pericarditis

eTable 2. Crude Rate of Reported Myocarditis Meeting BC Level 1 and 2 Only per Million Doses Administered and 95% CI by Vaccine Product, Dose Number, Age, and Sex: Series Initiated on or After June 1, 2021

eTable 3. Crude Rate of Reported Myocarditis and Pericarditis per Million Doses Administered and 95% CI by Vaccine Product, Dose Number, Age, and Sex: Series Initiated on or After December 14, 2020

eFigure 3. Rate of Reported Myocarditis and Pericarditis per Million Doses Administered and 95% CI Following Dose 2 of mRNA Vaccine for All Sexes in Ontario as of September 4, 2021

eTable 4. Rate of Reported Myocarditis and Pericarditis per Million Doses Administered and 95% CI by Dose 2 Product and Interdose Interval, for Individuals Receiving Dose 2 on or After June 1, 2021

eTable 5. Rates of Reported Myocarditis and Pericarditis per Million Doses Administered and 95% CI Among Males Aged 18-24 Years by Vaccine Product and Interdose Interval With Dose 2 on or After June 1, 2021

This supplementary material has been provided by the authors to give readers additional information about their work.

© 2022 Buchan SA et al. *JAMA Network Open*.

eFigure 1. Number of COVID-19 mRNA vaccine doses administered in Ontario by dose number and vaccine product and overview of vaccine program changes

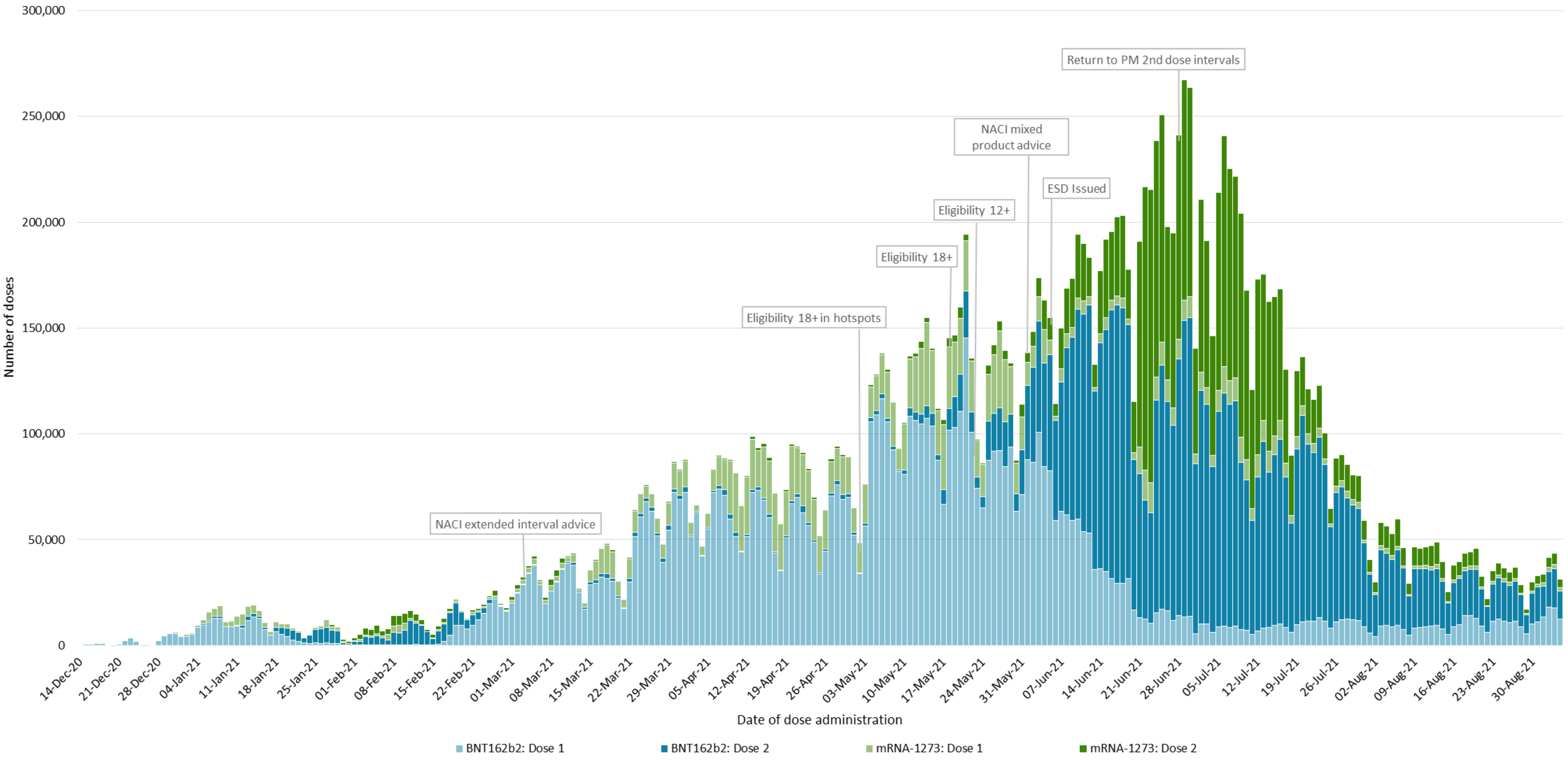

NACI: National Advisory Committee on Immunization; ESD: Enhanced Surveillance Directive; PM: Product Monograph

© 2022 Buchan SA et al. *JAMA Network Open*.

eFigure 2. Myocarditis and pericarditis reports following COVID-19 mRNA vaccines by dose number and time to symptom onset[a]

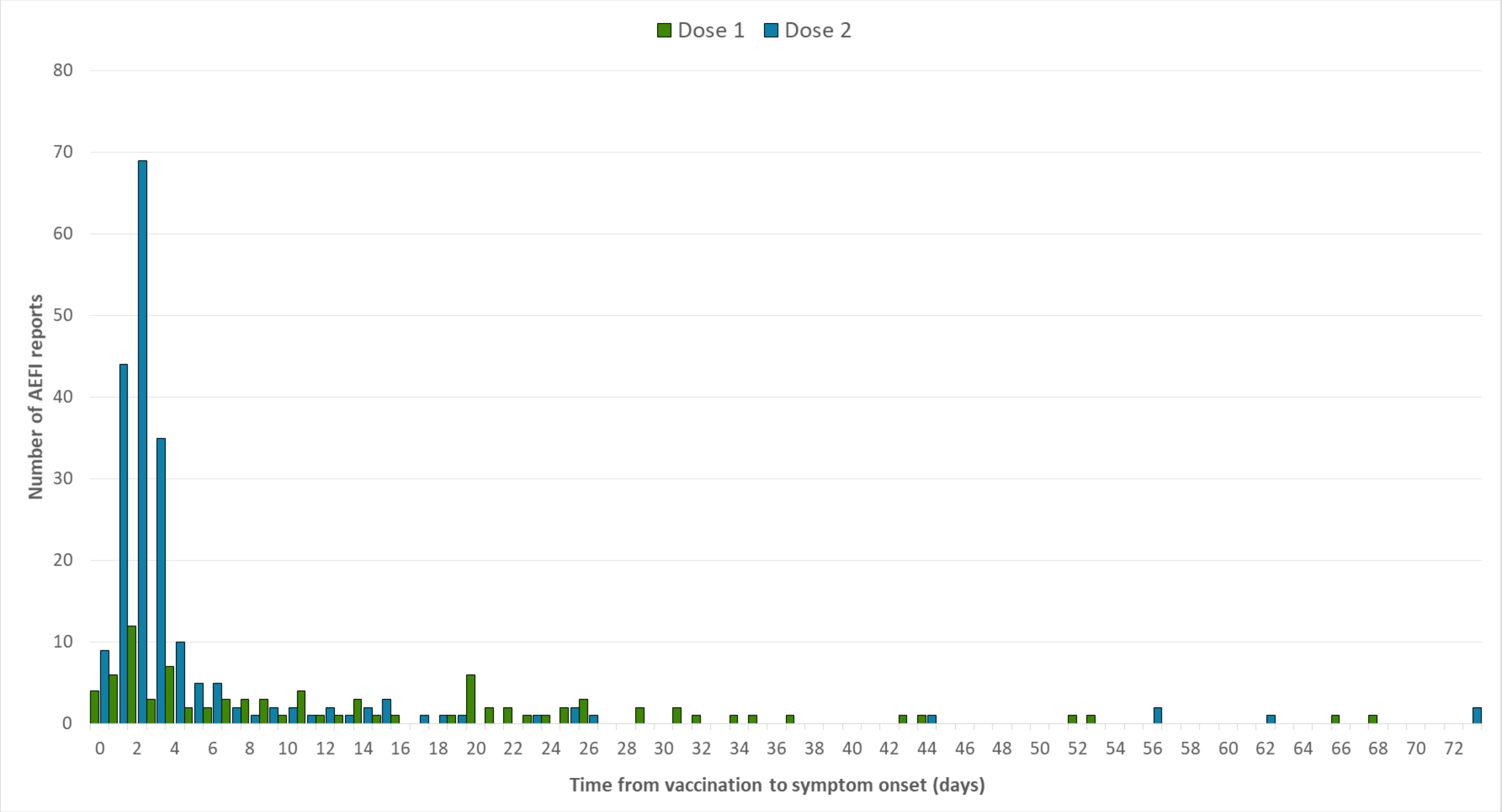

[a]2 reports with unknown time to onset were excluded from this figure

© 2022 Buchan SA et al. *JAMA Network Open*.

eTable 1. Clinical diagnosis and severity of myocarditis and pericarditis

	**Myocarditis (n=105)**	**Pericarditis (n=85)**	**Myopericarditis[a] (n=107)**	**Total (N=297)**
**Age group (years)**				
12-17	28 (26.7%)	8 (9.4%)	19 (17.8%)	55 (18.5%)
18-24	30 (28.6%)	16 (18.8%)	50 (46.7%)	96 (32.3%)
25-39	30 (28.6%)	12 (14.1%)	30 (28.0%)	72 (24.2%)
≥40	17 (16.2%)	49 (57.6%)	8 (7.5%)	74 (24.9%)
**Healthcare utilization/outcome**				
Emergency department	101 (96.2%)	83 (97.6%)	106 (99.1%)	290 (97.6%)
In-patient hospitalization	87 (82.9%)	33 (38.8%)	90 (84.1%)	210 (70.7%)
Intensive care unit admission	5 (4.8%)	5 (5.9%)	4 (3.7%)	14 (4.7%)
Death	0	0	0	0
**BC level**				
1	34 (32.4%)	5 (5.9%)	28 (26.2%)	67 (22.6%)
2	68 (64.8%)	73 (85.9%)	79 (73.8%)	220 (74.1%)
3	3 (2.9%)	7 (8.2%)	0 (0.0%)	10 (3.4%)

[a]Myopericarditis reports were assessed against Brighton Collaboration (BC) case definitions for myocarditis and pericarditis. The highest level of certainty was assigned to the report (e.g., if a report met level 1 for myocarditis and level 3 for pericarditis, it was assigned as level 1).

© 2022 Buchan SA et al. *JAMA Network Open*.

eTable 2. Crude rate[a] of reported myocarditis meeting BC level 1 and 2 only per million doses administered and 95% CI by vaccine product, dose number, age, and sex: series initiated on or after June 1, 2021

**BNT162b2**						
	All		Female		Male	
**Age group (years)**	Dose 1	Dose 2	Dose 1	Dose 2	Dose 1	Dose 2
**12-17**	21.5 (10.7 - 38.4)	49.7 (30.8 - 76.0)	8.1 (1.0 - 29.1)	9.7 (1.2 - 35.1)	34.2 (15.6 - 64.9)	88.1 (53.0 - 137.5)
**18-24**	10.7 (2.2 - 31.3)	19.0 (3.9 - 55.5)	7.9 (0.2 - 44.1)	NR	13.1 (1.6 - 47.3)	35.5 (7.3 - 103.7)
**25-39**	9.3 (3.0 - 21.7)	12.8 (3.5 - 32.9)	NR	13.1 (1.6 - 47.5)	17.9 (5.8 - 41.8)	12.6 (1.5 - 45.4)
**≥40**	NR	NR	NR	NR	NR	NR
**Total**	10.2 (6.1 - 15.9)	23.2 (15.4 - 33.5)	3.4 (0.7 - 9.8)	6.8 (1.9 - 17.4)	16.6 (9.5 – 27.0)	38.8 (24.9 - 57.8)
**mRNA-1273**						
	All		Female		Male	
**Age group (years)**	Dose 1	Dose 2	Dose 1	Dose 2	Dose 1	Dose 2
**12-17[b]**	NA	NA	NA	NA	NA	NA
**18-24**	NR	195.5 (117.7 - 305.3)	NR	69.1 (14.2 - 201.9)	NR	299.5 (171.2 - 486.4)
**25-39**	16.2 (3.3 - 47.3)	48.9 (23.5 - 90.0)	NR	21.5 (2.6 - 77.7)	28.8 (5.9 - 84.3)	72.1 (31.1 - 142.0)
**≥40**	10.0 (1.2 - 36.1)	NR	NR	NR	18.3 (2.2 - 66.2)	NR
**Total**	10.4 (3.4 - 24.4)	58.4 (39.1 - 83.9)	NR	22.0 (7.1 - 51.4)	18.7 (6.1 - 43.7)	89.4 (57.3 - 133.0)

[a]Estimates were not provided for strata with 0 reported events. [b]Estimates for mRNA-1273 were not provided for individuals aged 12-17 because this product was not used for this age group in Ontario. NA, not applicable; NR, not reported.

© 2022 Buchan SA et al. *JAMA Network Open*.

eTable 3. Crude rate of reported myocarditis and pericarditis per million doses administered and 95% CI by vaccine product, dose number, age, and sex: series initiated on or after December 14, 2020

BNT162b2						
	All		Female		Male	
**Age group (years)**	Dose 1	Dose 2	Dose 1	Dose 2	Dose 1	Dose 2
**12-17**	24.7 (14.9 - 38.6)	53.6 (37.5 - 74.2)	15.9 (5.8 - 34.6)	12.0 (3.3 - 30.8)	33.4 (17.8 - 57.0)	94.5 (64.6 - 133.4)
**18-24**	18.8 (10.5 - 31.1)	24.3 (13.0 - 41.6)	7.5 (1.5 - 21.8)	7.2 (0.9 - 25.9)	30.6 (15.8 - 53.5)	43.4 (21.7 - 77.6)
**25-39**	8.2 (4.6 - 13.4)	13.2 (7.7 - 21.2)	5.3 (1.7 - 12.3)	11.7 (5.0 – 23.0)	11.3 (5.4 - 20.7)	15.0 (6.9 - 28.5)
**≥40**	4.2 (2.5 - 6.5)	6.4 (4.2 - 9.5)	2.0 (0.7 - 4.7)	4.7 (2.3 - 8.7)	6.8 (3.7 - 11.3)	8.5 (4.8 - 14.1)
**Total**	8.5 (6.6 - 10.8)	14.3 (11.5 - 17.5)	4.5 (2.7 - 7.1)	7.0 (4.5 - 10.5)	13.1 (9.7 - 17.3)	22.7 (17.6 - 28.8)
**mRNA-1273**						
	All		Female		Male	
**Age group (years)**	Dose 1	Dose 2	Dose 1	Dose 2	Dose 1	Dose 2
**12-17**[b]	NA	NA	NA	NA	NA	NA
**18-24**	16.2 (4.4 - 41.4)	177.8 (136.9 - 227.1)	17.4 (2.1 - 63.0)	45.7 (19.7 – 90.0)	15.2 (1.8 - 54.8)	305.2 (230.6 - 396.4)
**25-39**	10.5 (3.9 - 22.9)	39.1 (27.1 - 54.6)	7.5 (0.9 - 27.1)	12.1 (3.9 - 28.2)	13.3 (3.6 - 33.9)	63.9 (42.8 - 91.8)
**≥40**	10.1 (5.2 - 17.7)	8.3 (4.9 - 13.2)	8.2 (2.7 - 19.1)	3.7 (1.0 - 9.4)	12.3 (4.9 - 25.3)	13.2 (7.2 - 22.1)
**Total**	11.0 (6.9 - 16.6)	34.2 (28.3 - 41)	9.1 (4.1 - 17.2)	10.1 (5.9 - 16.2)	12.9 (6.9 - 22.1)	58.2 (47.3 - 70.9)

[a]Estimates were not provided for strata with 0 reported events. [b]Estimates for mRNA-1273 were not provided for individuals aged 12-17 because this product was not used for this age group in Ontario. NA, not applicable; NR, not reported.

© 2022 Buchan SA et al. *JAMA Network Open*.

eFigure 3. Rate of reported myocarditis and pericarditis per million doses administered following dose 2 of mRNA vaccine for all sexes in Ontario as of September 4, 2021

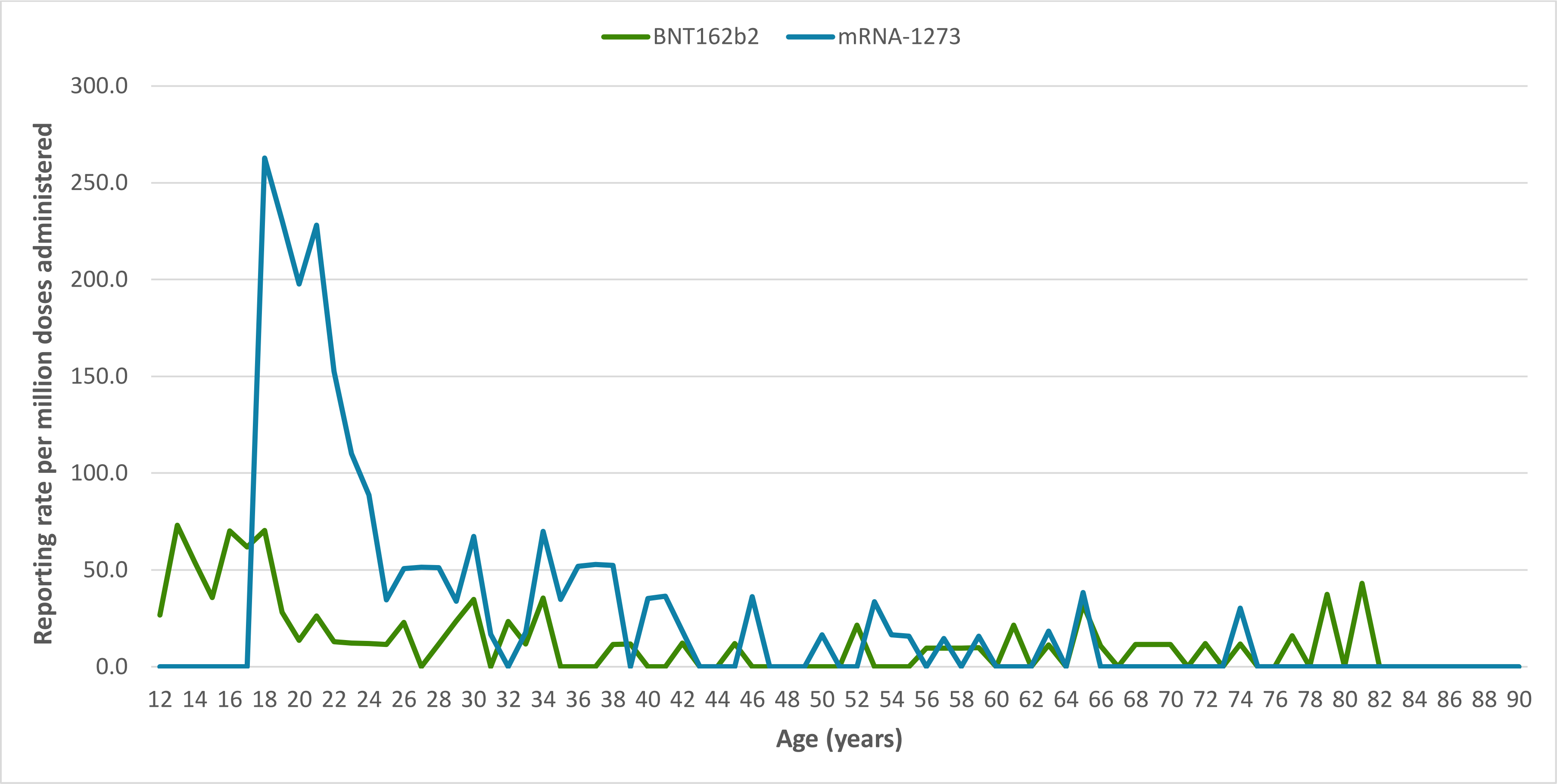

© 2022 Buchan SA et al. *JAMA Network Open*.

eTable 4. Rate[a] of reported myocarditis and pericarditis per million doses administered and 95% CI by dose 2 product and interdose interval, for individuals receiving dose 2 on or after June 1, 2021

**BNT162b2 – Dose 2**							
	Females	Males	12-17 years[c]	18-24 years	25-39 years	≥40 years	OVERALL
**Product schedule*[b]**							
BNT162b2-BNT162b2	5.9 (3.5 - 9.5)	25.0 (19.3 - 32.0)	53.8 (37.7 - 74.5)	26.9 (14.3 - 45.9)	13.4 (7.5 - 22.1)	5.4 (3.1 - 8.6)	14.9 (11.9 - 18.6)
mRNA-1273 – BNT162b2	NR	14.6 (0.4 - 81.2)	NA	NR	NR	12.5 (0.3 - 69.7)	7.6 (0.2 - 42.3)
**Interval (days)**							
≤30	25.8 (8.4 - 60.3)	79.3 (44.4 - 130.8)	101.9 (55.7 - 170.9)	45.3 (5.5 - 163.7)	42.5 (11.6 - 108.7)	NR	52.1 (31.8 - 80.5)
31-55	4.1 (1.1 - 10.6)	28.2 (18.6 - 41.0)	37.7 (21.6 - 61.3)	34.7 (15.9 - 66)	8.7 (2.8 - 20.3)	1.5 (0.0 - 8.3)	16.1 (10.9 - 22.8)
≥56	4.5 (2.0 – 9.0)	15.6 (9.9 - 23.4)	55.7 (20.4 - 121.2)	10.1 (1.2 - 36.5)	12.3 (4.5 - 26.7)	6.9 (4.0 - 11.1)	9.6 (6.5 - 13.6)
**mRNA-1273 – Dose 2**							
	Females	Males	12-17 years**	18-24 years	25-39 years	≥40 years	OVERALL
**Product schedule**							
mRNA-1273 - mRNA-1273	9.5 (3.8 - 19.6)	58.3 (42.4 - 78.3)	NA	162.0 (108.5 - 232.6)	30.1 (16.0 - 51.4)	10.2 (4.7 - 19.4)	34.2 (25.4 - 44.9)
BNT162b2 -mRNA-1273	13.1 (6.0 - 25.0)	72.5 (53.8 - 95.6)	NA	203.9 (142.0 - 283.6)	52.0 (32.2 - 79.5)	3.8 (0.8 – 11.0)	42.9 (32.6 - 55.3)
**Interval (days)**							
≤30	36.2 (7.5 - 105.8)	125.7 (64.9 - 219.5)	NA	353.1 (182.4 - 616.8)	39.5 (8.1 - 115.4)	NR	83.9 (47.0 - 138.4)
31-55	9.4 (3.5 - 20.5)	96.0 (74.4 - 121.9)	NA	184.0 (133.7 - 247.0)	45.0 (29.1 - 66.4)	7.4 (2.0 – 19.0)	54.6 (42.8 - 68.7)
≥56	10.0 (4.0 - 20.6)	23.1 (12.9 – 38.0)	NA	103.2 (44.5 - 203.3)	29.4 (10.8 - 64)	7.5 (3.2 - 14.7)	16.2 (10.2 - 24.6)

[a]Estimates were not provided for strata with 0 reported events. [b]There were 5 reports following ChAdOx1-mRNA-1273 among 350,628 doses administered and 5 reports following ChAdOx1-BNT162b2 among 279,805 doses administered. These are excluded from Table 5.[c]Estimates for mRNA-1273 were not provided for individuals aged 12-17 because this product was not used for this age group in Ontario. NA, not applicable; NR, not reported.

© 2022 Buchan SA et al. *JAMA Network Open*.

eTable 5. Rates[a] of reported myocarditis and pericarditis per million doses administered and 95% CI among males aged 18-24 years by vaccine product and interdose interval with dose 2 on or after June 1, 2021

Vaccine schedule	Reports (N)	Doses administered (N)	**Reported rate (95% CI) per million doses**
People with two doses			
Homologous schedule			
BNT162b2-BNT162b2	11	235,819	46.6 (23.3 – 83.5)
Interval ≤30 days	2	21,160	94.5 (11.4 – 341.4)
Interval 31-55 days	8	124,235	64.4 (27.8 – 126.9)
Interval ≥56 days	1	90,424	11.1 (0.3 – 61.6)
mRNA-1273-mRNA-1273	27	93,616	288.4 (190.1 – 419.6)
Interval ≤30 days	4	10,623	376.5 (102.6 – 964.1)
Interval 31-55 days	20	60,352	331.4 (202.4 – 511.8)
Interval ≥56 days	3	22,641	132.5 (27.3 – 387.2)
Heterologous schedule			
mRNA-1273-BNT162b2	0	8,853	NR
Interval ≤30 days	0	1,058	NR
Interval 31-55 days	0	5,402	NR
Interval ≥56 days	0	2,393	NR
BNT162b2-mRNA-1273	29	85,893	337.6 (226.1 - 484.9)
Interval ≤30 days	6	7,720	777.2 (285.2 - 1691.6)
Interval 31-55 days	20	62,717	318.9 (194.8 - 492.5)
Interval ≥56 days	3	15,456	194.1 (40.0 - 567.2)

[a] Estimates were not provided for strata with 0 reported events. NR, not reported.

© 2022 Buchan SA et al. *JAMA Network Open*.
